# The next generation of biopanning: next gen sequencing improves analysis of bacterial display libraries

**DOI:** 10.1186/s12896-019-0577-8

**Published:** 2019-12-21

**Authors:** Sarah D. Stellwagen, Deborah A. Sarkes, Bryn L. Adams, Mia A. Hunt, Rebecca L. Renberg, Margaret M. Hurley, Dimitra N. Stratis-Cullum

**Affiliations:** 1 Biotechnology Branch, CCDC US Army Research Laboratory, 2800 Powder Mill Rd, Adelphi, 20783 MD USA; 2 Department of Biological Sciences, University of Maryland Baltimore County, 1000 Hilltop Circle, Baltimore, 21250 MD USA; 3 General Technical Services, Suite 301, 1451 Route 34 South, Wall Township, 07727 NJ USA

**Keywords:** Bacterial display, Directed evolution, Peptide display, Biopanning, NGS

## Abstract

**Background:**

Bacterial surface display libraries are a popular tool for novel ligand discovery due to their ease of manipulation and rapid growth rates. These libraries typically express a scaffold protein embedded within the outer membrane with a short, surface-exposed peptide that is either terminal or is incorporated into an outer loop, and can therefore interact with and bind to substrates of interest.

**Results:**

In this study, we employed a novel bacterial peptide display library which incorporates short 15-mer peptides on the surface of *E. coli*, co-expressed with the inducible red fluorescent protein DsRed in the cytosol, to investigate population diversity over two rounds of biopanning. The naive library was used in panning trials to select for binding affinity against 3D printing plastic coupons made from polylactic acid (PLA). Resulting libraries were then deep-sequenced using next generation sequencing (NGS) to investigate selection and diversity.

**Conclusions:**

We demonstrated enrichment for PLA binding versus a sapphire control surface, analyzed population composition, and compared sorting rounds using a binding assay and fluorescence microscopy. The capability to produce and describe display libraries through NGS across rounds of selection allows a deeper understanding of population dynamics that can be better directed towards peptide discovery.

## Background

Natural selection has resulted in elegant solutions to the pressures that organisms face. On a macro scale, examples include the cryptic shape and coloration of stick insects, or the specialized feeding organs of mosquitoes, and microscopically, ligand binding and enzymes. Artificially harnessing the power of natural selection has not only provided useful organisms at a larger scale that have shaped culture and diet (e.g. agricultural varieties of plants and animals), but has also contributed to discovering molecular tools that have influenced disciplines from medicine to materials research. Selection of particular molecular interactions is key to natural and engineered antibody-antigen recognition for diagnostics, new materials, and an understanding of chemical interactions [1–4]. Peptide discovery that exploits artificial selection pressures to shape diverse peptide libraries have led to a wide variety of unique sequences for targeted use [5–9]. Understanding library diversity, how peptide populations evolve during the discovery process, as well as methods to analyze diversification on a deeper scale are important for improving discovery techniques that rapidly produce unique molecules for purpose-driven benefit.

Surface display libraries employ polypeptides (including peptides, scaffold proteins, and antibody fragments) typically presented at the surface of phage coats or bacterial, yeast, or mammalian cell membranes. Each organism expresses a unique, random sequence with the potential to interact with specific antigenic or molecular targets [10]. After incubating a library with a substrate of interest, the unbound members are washed away while the remaining bound members are amplified and used for further screening under more stringent conditions or analyzed to understand binding interactions. While phage display has been the traditional system used for ligand screening, bacterial display libraries provide advantages due to ease of manipulation, as phage reinfection steps are eliminated, and fast growth rates, which are important factors for rapid discovery of binding peptides to assess new and emergent threats [6, 8, 9].

**Fig. 1:**
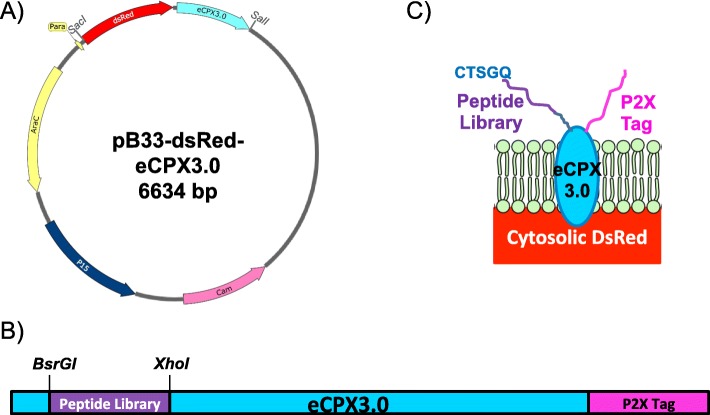
pB33-dsRed-eCPX3.0 Peptide Library Design. **a** Plasmid map of DsRed-peptide display expression plasmid showing relative location and orientation of genes. **b** Diagram of eCPX3.0 gene from 5’ to 3’ indicating location of 15-mer peptide display library sequence, restriction sites flanking the library, and the location of the C-terminal P2X tag. **c** Schematic of mature eCPX3.0 display scaffold embedded in the cell membrane with N-terminal peptide library (preceded by CTSGQ) and C-terminal P2X tag displayed at the cell surface, and DsRed accumulating in the cytosol for cell visualization. The N-terminal peptide library contains random sequences, while the fixed C-terminal P2X tag allows expression monitoring through binding to the fluorescent protein YPet-Mona

**Fig. 2:**
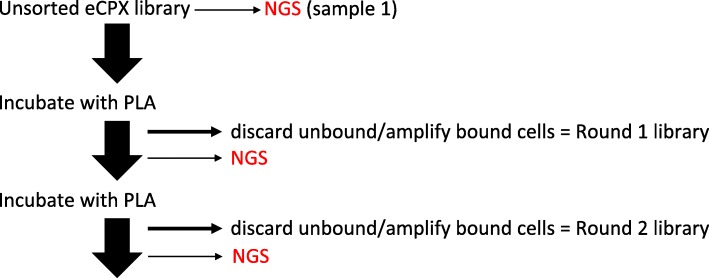
Flow Chart for Biopanning and NGS Sequencing. The naive, unsorted library was both sequenced and used for biopanning against PLA 3D printing plastic. After washing the PLA sample and amplifying bound library members, the Round 1 library was sequenced and again panned against fresh PLA. The final, Round 2 library was sequenced along with the naive and Round 1 libraries

**Fig. 3:**
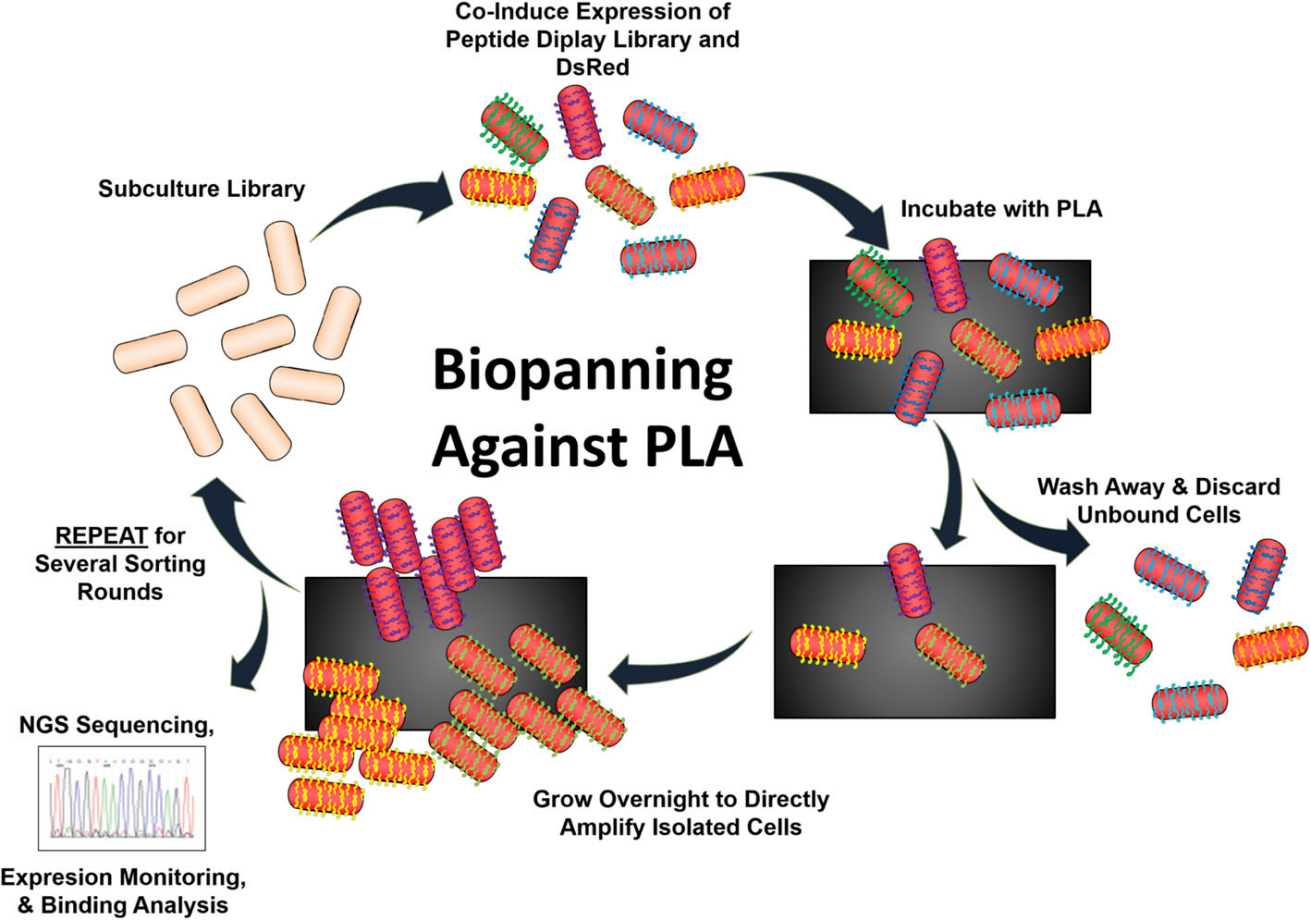
Biopanning experimental design schematic. Biopanning is a cyclical process that begins with subculturing the naive, unsorted library. Co-expression of cytosolic DsRed and membrane-localized peptides from the pB33-dsRed-eCPX3.0 library is achieved by induction with L-arabinose during log phase of cell growth. The induced library is then incubated with PLA 3D printing plastic for 15 min with shaking. Next, the PLA coupons were washed vigorously with PBS 1% Tween for 30 min to remove unbound cells, and then placed in LB +Cm with 2% D-Glucose media to proliferate and recover the bound library. The presence of glucose halts the production of new copies of membrane-displayed peptides, which are slowly diluted during cell proliferation, along with the cytosolic DsRed. The process is repeated for sorting rounds 1 and 2, changing only the starting library material (using the previous sorting round). After each round, NGS sequencing was performed and peptide expression level monitored. Bound cells can be visualized directly on the PLA using fluorescence microscopy due to the presence of DsRed, and binding level can also be indirectly compared using regrowth assays and cell counting

Historically, peptide libraries were assumed to be highly diverse, unbiased sequence compilations ready for selection against a target material, and examination of the enriched peptides was limited by Sanger sequencing of a few hundred sequences. High throughput next generation sequencing (NGS) technologies now provide an avenue to investigate populations at a much broader scale [11–13]. We use a newly generated library (Fig. 1) to assess diversity and follow population evolution across rounds of biopanning against polylactic acid (PLA) 3D printing plastic. This library relies on a previously engineered outer membrane scaffold protein (eCPX) that co-presents random 15-mer peptides for screening on the N-terminus, and a fixed P2X peptide at the C-terminus to monitor expression via binding to the fluorescent protein YPet-Mona [14–16]. If a stop codon were present in the random peptide, P2X binding by YPet-Mona (Fig. 1b and c) would not occur because the remainder of the eCPX scaffold would not be produced or shuttled to the outer membrane. This interaction can be monitored using Fluorescence-Activated Cell Sorting (FACS) [17]. Additionally, we incorporated inducible DsRed fluorescence into the vector cassette (Fig. 1) allowing visualization to compare binding efficiency as an alternative method to indirect binding assays [6]. Indirect binding assays measure the cell proliferation of bound cells after a recovery stage to extrapolate and relatively quantify the number of bound library members. Serial dilution and plating of the resulting culture of amplified cells allows counting of colony forming units, while visualizing and quantifying cells directly bound to nonconductive materials by scanning electron microscopy (SEM) would require sputter coating with a metal such as gold. Here, we analyzed peptide sequences from the naive library and after each round of biopanning using Illumina NGS technology (Fig. 2) to understand the overall dynamics of population selection, and then visualized the bound cells directly on the PLA surface using the incorporated DsRed protein with fluorescence microscopy. The biopanning process is described in detail in Fig. 3, and is similar to previously described methods for isolating aluminum binders using a related eCPX-based library [6], except that here we additionally co-expressed the DsRed protein for direct visualization.

## Results

### Analysis of library diversity

**Fig. 4:**
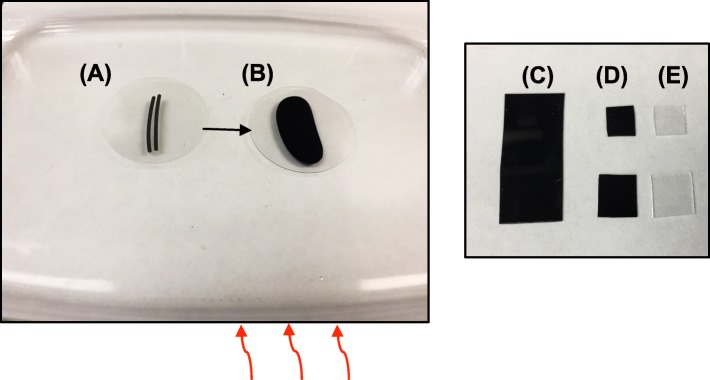
Polylactic Acid (PLA) and sapphire material sample preparation. Two pieces cut from a spool of PLA printing filament ∼4 cm in length were placed on a Pyrex baking dish under a sapphire wafer (**a**) and then heated with a hot plate until melted (**b**). After melting, PLA pieces were removed from the Pyrex dish and sapphire wafers, then cut into coupons for biopanning trials (**c**), or cut into smaller pieces used for binding quantification (**d**) with equally sized sapphire pieces (**e**)

**Fig. 5:**
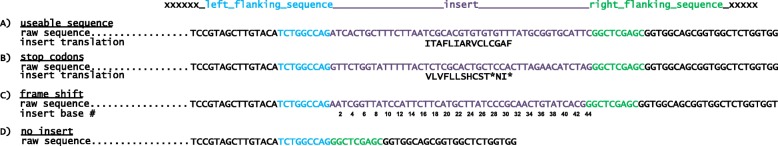
Strategy for filtering NGS sequences. A valid insert sequence consists of correct flanking regions, an insert within the correct reading frame (typically 45 bp), and without stop codons (**a**). Sequences that translate into an insert with stop codons (**b**), result in a frame shift (**c**), or that have no insert (**d**) are filtered into separate categories

**Fig. 6:**
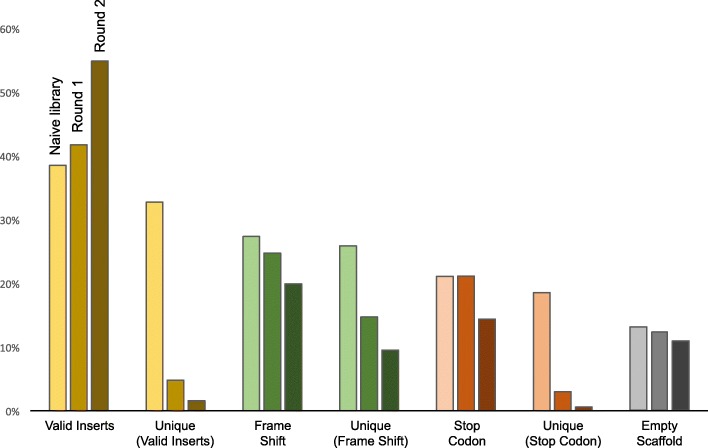
Percent representation of each insert type from the naive and sorted libraries after NGS sequencing. Colors are grouped by total representation of the library followed by frequency of each unique insert for each category, and are in the order Naive library, Round 1, and Round 2. The Empty Scaffold category does not have a frequency representation, as members cannot be tracked across panning rounds. Raw sequence counts can be found in Additional file 1: Table S1

We used Illumina next generation sequencing technology to assess peptide diversity for a newly created bacterial display library and after 2 rounds of biopanning against PLA 3D printing plastic (Figs. 2 and 3). The PLA coupons were prepared by melting filaments together as described in Fig. 4 and the experimental methods. Illumina NGS sequencing resulted in 1,212,302 reads from the naive library, 1,418,018 reads from the Round 1 library, and 1,405,367 reads from the Round 2 library (Additional file 1: Table S1). The strategy for filtering sequences from this data is outlined in Fig. 5, showing examples of valid, useable sequence, and invalid sequences containing stop codons, frame shifts, or no peptide insert. We define ’unique’ sequences as the total complement of different amino acid sequences for any category. For example, a valid insert with more than one representative is counted as a single representative of the ’unique’ category for valid sequences. Valid inserts (inserts in the correct reading frame without stop codons) comprised 38% of all sequences in the naive library, 85% of which were unique (or 33% of the total library; Fig. 6). After one round of selection, valid inserts increased to represent 42% of the library population; however, 11% of these (5% of the total library) were unique. The second round of sorting resulted in more than half the library’s representation as valid inserts (55%), with an even further decrease in unique valid inserts (3% of valid inserts were unique or 2% of the total library). Library members with inserts that resulted in a frame shift, contained stop codons, or that had an empty scaffold made up 27%, 21%, and 13% of the naive library population, respectively. A general reduction was observed for members from these categories across panning rounds, accompanied by the distinct reduction in the representation of unique individuals, as compared to valid sequences (Fig. 6).

The most frequent sequences represented in the naive, unsorted library generally consisted of single-residue inserts (Table 1), although the bulk of the unsorted library consisted of 15-mers (375,287 out of 396,641 unique sequences, or 95%). Leucine alone had the highest representation and was identified 32 times (0.003%), while the most frequent 15-mer (PRKTLKGTLTVPSYI) was identified 8 times (0.0007%). The most common insert sequence discovered in the Round 1 library, a 15-mer (VLPQTSFFAATCRRS), was represented 115 times (0.01%), which increased to 283 in Round 2. 15-mers were again the main component of the library in Round 1, consisting of 64,507 out of 67,885 sequences (95%). The most common insert sequence discovered in Round 2, also a 15-mer (CHISPEKRRLIVCAD), was represented 4,619 times (0.38%), which increased from 28 in Round 1. In Round 2, 15-mers comprised 20,903 of 21,931 unique sequences (95%). The frequency of the population’s top sequence increased 4-fold from the naive library to Round 1, and 40-fold from Round 1 to Round 2.

**Table 1:** Top 40 most frequent sequences from the naive library and after 2 rounds of biopanning

	Naive Library	No. Seqs	Round 1	No. Seqs	Round 2	No. Seqs
1	L	32	VLPQTSFFAATCRRS	115	CHISPEKRRLIVCAD	4619
2	P	31	SNRLRGENLTFRRHK	110	YPLCSTYGNVLVGGS	3989
3	R	30	DRRRKIERYDAQISV	89	PLKQGIYETTSTKGT	3321
4	S	22	T	87	YGSRTFPVCSTVHRR	2185
5	A	19	S	84	YRLMEMEEYHKDVFA	1985
6	T	13	PAKGSIKNPILLGIR	74	KHLLCVMQGGTAFRG	1783
7	E	12	QPLSESLEDKTSIDV	74	RIFGFLSCKPNIIKL	1094
8	H	11	SS	73	IVNRMWCLISVISQA	921
9	SP	11	THNARRLMPCIDASD	73	FWGLYPLFCCTGCSP	909
10	Y	11	YPGRMEALFAHVSNA	73	SKLFSSWVLCACWHC	778
11	Q	10	RDPSWRAETVNCGNL	69	AVLAIYPNSLYWLQA	656
12	TT	10	RHQNPQEEI	69	YIAMILHGFSQAFPG	650
13	V	10	YTYHGRNSERLLRMK	69	LLFSFFNCFTGNDGQ	636
14	F	9	TQDETRRGGMSTTSS	68	NKWSPRTDFNTESVR	623
15	GPCLETCLTA	9	HTYCVHPSMATCWHY	66	HAPWECLISVLASAH	616
16	ERSVG	8	ANLPDSHHIAHNKDT	65	ARGLLTAISFYAFQC	602
17	LT	8	AMPDFVILDHGVTRR	64	RLWMRSPTSTAGGIR	601
18	PRKTLKGTLTVPSYI	8	KNSLTCCYG	63	AERLHQITVMLLRSF	595
19	SG	8	RRNYIHIGAKAAGRS	63	LSNLLHYLYALGQPG	595
20	SKAPLRKTNMKLR	8	DVLAKYSSLERQDLP	62	PILLCYVLYCEYASS	587
21	TS	8	SVAATCWRYWYDKPL	62	SWTMLICQLHSYLYT	583
22	ATYYLQPYVPISEET	7	RPPCEEMSINCPGVT	61	PRALWEASMVVTLAC	581
23	DQLGVL	7	TYPCIYA	61	AVCKPVGPKAPWRDR	575
24	GPWATWSSYDKWHNR	7	VSLIDQCDCTMQSNP	61	SSRSLQQNSISRLSM	571
25	GT	7	HRRRKPIHLPEYPLP	60	IRKQWDFLCLGFLLS	559
26	KS	7	RHSICPRFRGLLPHS	60	SAFPACGLASYFPWF	554
27	PCNFISKHFVPHRRH	7	RML	60	GVVRHCPAWQKSPWP	532
28	PIVGVYK	7	ARTSG	59	HSALDVLSWVISSLM	532
29	PR	7	DWMKFHVEA	59	GLRIQAYALCYWEPV	526
30	SFLL	7	GPANWTCAWQAVTAF	59	NRAIIGEIAGLIKFL	522
31	TD	7	LLVKLGP	59	PAAAFFTQVMSLLRQ	503
32	WD	7	RGVGTSHPQSVCVKP	59	RLHCLERYINLAFLP	503
33	YT	7	CIT	58	RMHHLMFRDSHSHTD	502
34	AMTSLPSTCRQLHSN	6	WLHGVLLNFKKHGVC	58	TQKHKTLCFSIRKPG	494
35	AQDRVRLKSFGRASL	6	IHSAGRMSINLCVGI	57	LRYGIWQVSHIALTS	492
36	CQFNVWANLARIAIG	6	IRRTLLGRPVWFFEE	57	QSFVMESVLSFGFSF	492
37	EDGPTRLWPLSRAAW	6	RSSRSVDPLTYRRNA	57	DRRRKIERYDAQISV	486
38	FSLTVFVMDQENVNR	6	YQPWREDRRSSAGSA	57	PHKLWRLGCFVAMDL	485
39	GDSEGLRMSTLGSSS	6	GLVRVFWPTPERGHR	56	KTVFCLTVLPRFEAA	482
40	GTIGFLDLPRKTVMR	6	AVKAYSTFRQSPTRL	55	RHLVSWLCTVLHPDP	481

Representation of all 20 amino acids as a decimal of the overall (i.e. all unique sequences and not just 15-mer) library character was generated round by round, both including and excluding frequency of sequence occurrence (Additional file 1: Figure S1). This represents a flat numeric analysis of current sequencing data with no correction for background probabilities from previous genomic analyses. Despite that, this information is useful as a depiction of both static and dynamic characteristics of the library. There is a consistently high representation of hydroxyl containing residues S, T, basic residue R, and hydrophobic residue L. Additionally there is underrepresentation from acidic residues D and E and hydrophilic residues N and Q. Round by round modifications to the distribution are subtle, but there is an increase in hydrophilic residues C and Y, an increase in hydrophobic residues M and L, an increase in aromatic residues F and W, and a decrease in D, E, N and Q. These trends are visible regardless of the incorporation of sequence frequency.

Analysis of sequence motifs in round by round 15-mer sequence character by the pLogo generator [18] further examines the inclusion of background probability statistics for *E. coli* K12 and provides an assessment of spatial organization within the sequence (Additional file 1: Figure S2). The trends seen from the rough numbers in Additional file 1: Figure S1 are accentuated in this representation. There is an up-regulation of hydrophilic residues S, T, C, Y, up-regulation of aromatic residue W and basic residue R, down-regulation of acidic residues D and E, and down-regulation of nonpolar residues A, G and L. The increase in M, C, and W by round is more visible in this representation, as is the down-regulation by round of E and A. By round 2, dominance of key residues C, W, S, M and R are clearly visible. There is very little spatial dependence on this up- (or down-) regulation, as befits interactions with a surface and with sequences which are largely expected to be loose coil in structure (as can be seen from the list in Table 1). There are further differences in sequence character in round 2 when comparing the pLogo generated motif of the entire 15-mer population of the library (Additional file 1: Figure S3).

The increase in cysteine after sorting is particularly interesting since it could have both positive and negative effects on the peptide library. Cysteine could help constrain any structural component of the peptide that aids in binding affinity or specificity by creating disulfide bonds that increase rigidity [19]. However, when several cysteine residues are present in the library, unwanted intermolecular disulfide bonding could also occur, which could cause clumping of the displayed cells and interfere with isolation of individual peptides within the population for characterization. The peptides fused by disulfide bonds could have cooperative binding effects, which is not a problem in and of itself, but could be difficult to understand and replicate off-cell. In this study it was shown that the percentage of peptide sequences with zero cysteines decreases round by round, and the percentage of sequences containing more than a single cysteine changes only slightly (Additional file 1: Table S2). Since the increase in cysteine is primarily due to an increase in peptides containing a single cysteine, intra-sequence disulfide binds are not significantly changing and probably don’t have much effect on peptide rigidity.

### Binding assays

**Fig. 7:**
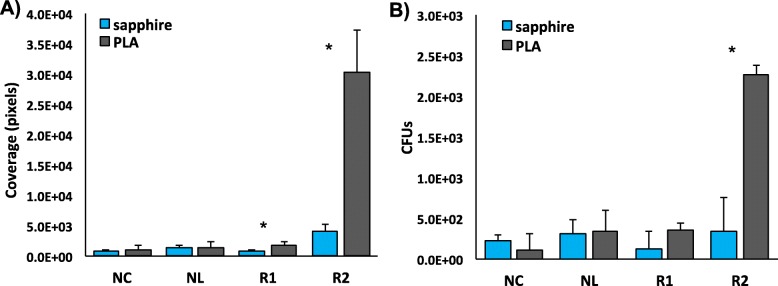
Binding quantification for each assay type. After incubation of libraries and negative control with PLA and sapphire, the number of red pixels were quantified using fluorescent microscopy (**a**) and colony forming units quantified using indirect binding methods (**b**). NC = negative control, NL = naive library, R1 = Round 1, R2 = Round 2. Mean ± standard error

**Fig. 8:**
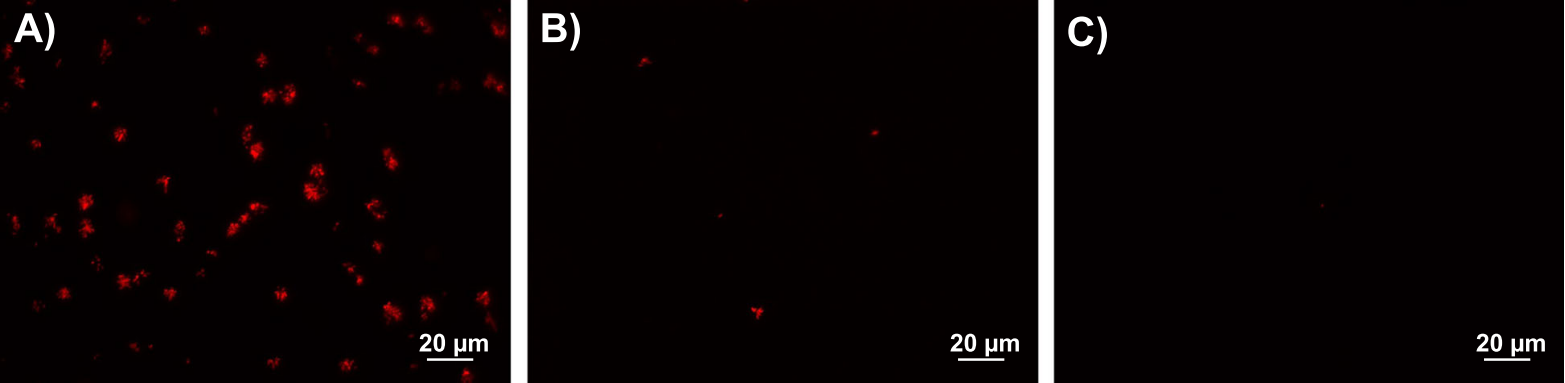
Fluorescent microscopy images of bound cells. **a** Round 2 library on PLA, **b** Round 2 library on sapphire wafer, and **c** negative control on PLA after 10 min incubation with sample materials and 10 min washing

We compared a binding assay using direct visualization that measures fluorescent pixel coverage of microscopy images (Figs. 7a & 8) with an indirect binding assay that quantifies plated CFUs to estimate library density (Fig. 7b). We incubated PLA and sapphire samples with the naive library, libraries produced after each round of sorting, and negative control cultures. Both assays showed similar trends, and binding was significantly higher on the PLA coupons than sapphire pieces after Round 2 for both visualization (P <0.0001) and indirect (P = 0.0015) assays, and after Round 1 using the visualization assay (P <0.0001). There were no differences between the naive library, sorting rounds, and negative control when comparing growth curves (Additional file 1: Figure S4).

Along with the quantified results in Fig. 7, the fluorescence microscopy images shown in Fig. 8 demonstrate that after two rounds of sorting against PLA, cells displaying peptides from the 15mer library are able to bind the desired material and remain bound after vigorous washing (Fig. 8a). Furthermore, the binding level is higher for PLA (Fig. 8a) as compared to binding to a sapphire wafer (Fig. 8b). The enrichment of binding for PLA over sapphire indicates that the peptides are not just "sticky" in general, but have demonstrated affinity for the material of interest. There is some evidence of cell clumping in Fig. 8a, which may be caused by intermolecular disulfide bonds, discussed above, as a consequence of enrichment for cysteine residues in the library. Additionally, a peptide-free negative control culture, which still displays the empty eCPX scaffold and produces cytosolic DsRed, shows very little binding (Figs. 7 and 8c), indicating that the enriched peptide library is responsible for binding, rather than being caused by either of these over-expressed proteins, or by the cells used to generate the library.

## Discussion

Next generation sequencing technologies allow a deeper understanding of peptide selection from display libraries. For the first time, we used NGS to analyze libraries that employ bacterial display across rounds of biopanning to enrich libraries for surface binding. We sequenced populations from a newly developed, unsorted library (naive = N), and after two rounds of selection (Round 1 = R1, Round 2 = R2) against PLA 3D printing plastic (Figs. 5 and 6). Sequencing results confirm population diversity of the naive library and a reduction in this diversity across panning rounds (Fig. 6). Visualization of the Round 2 library demonstrates enrichment of PLA binding peptides after selection (Figs. 7 and 8). Valid, unique inserts represented 33% of the total naive bacterial display library (Fig. 6), more than double what was observed after deep sequencing of a naive 7-mer phage display library produced using NNK saturation mutagenesis, which recovered only 15% of sequences with valid, potential binders [20]. The representation of valid inserts increased through successive rounds of biopanning (N: 38%, R1: 42%, R2: 55% of the total library); however, the frequency of individual representatives decreases dramatically after just the first round (N: 33%, R1: 5%, R2: 2% of the total library), as adherent members are carried forward while weakly bound members are washed away in increasingly stringent washing steps.

The total fraction of nonbinding library members that display an empty scaffold, or contain sequence that results in a frame shifts or stop codon decreased 3–7% from the naive library across rounds of sorting (Fig. 6). Interestingly however, the frequency of individual sequences that contain frame shifts or stop codons (we cannot track empty scaffold individuals across rounds as they lack unique insert sequences) decreased more dramatically, reducing the diversity of these sequences 16% (frame shift) or 18% (stop codon). It is expected that after applying selective binding pressure, some individuals exhibiting binding not related to peptides or the display scaffold, or that are carried over for other reasons that are less understood (i.e. genomic mutations, growth rate advantages, etc.), would be enriched to some extent due to the natural evolution process. However, unlike the overall population increase observed for individuals that display a valid peptide (Fig. 6), the representation of nonbinding library members does not increase (note binding increase with sorting rounds in Fig. 7), suggesting a stronger association of valid inserts with the PLA surface. Nonbinding library population diversity does exhibit a similar dramatic decrease as compared to valid insert population diversity.

The library used in this study was created to reduce stop codons by employing 15 x NNS saturation mutagenesis, where N represents any base and S represents G or C. This technique produces a library of inserts with a representation of all 20 amino acids, but with an incidence rate of 3% of the codons in the naive library as stops. With 15-mer insert lengths, this translates to an expected 45% of sequences containing stop codons if stop codons occur singularly within an insert. Only 24% percent of our naive library scaffolds with a 15-mer contain at least one stop codon; a further 20% of these contain at least 2 or more stops. Moreover, a percentage of inserts with frame shifts (27% of the total library) also must contain stop codons, although it is not possible to distinguish a correct reading frame in order to quantify their frequency. There is a tradeoff between insert length and stop codon occurrence, as longer inserts with more residues have a higher chance of incorporating a stop. For example, libraries with a 7-mer insert length would be expected to have only a 21% stop codon occurrence. Library generation must balance the benefits of longer inserts that present secondary structure for enhanced binding with maintaining a diverse library of valid inserts free of stop codons.

The amino acid representation within the library does show subtle changes round by round as expected (Additional file 1: Figures S1 and S2). However, we note that the overall shape of the profile stays roughly the same (Additional file 1: Figure S1) and that diversity may be diminished but not extinguished (Fig. 6, Unique Valid Inserts). There is a general increase in residues capable of hydrogen bond donation consistent with the fact that PLA is a chemically generic aliphatic polyester capable of hydrogen bond acceptance through the polymer backbone. We also note an increase in the sulfur containing residues cysteine and methionine. n →π* interactions have been noted between cysteine sulfurs and protein backbone carbonyl groups [21] and similar interactions with the PLA backbone may contribute to the up-regulation of C and M clearly shown in the motif representation. Trends shown from the entire library to the top 40 most frequent sequences to the top 10 most frequent sequences in round 2 are also subtle. It is clear that the predominance of W in the library as a whole is less important in the context of the top 10 PLA binding sequences, while the cysteine interactions mentioned above are clearly in play for all. No true spatial dependence is flagged in any sequence analysis. We note from visual inspection of the top 10 binding sequences in Round 2 that hydrogen bond donors such as R, C, S, Y and T are highly represented.

Assessing populations for binding enrichment on non-antigenic material surfaces using bacterial display libraries in the absence of fluorescence has depended on methods that require additional steps, such as SEM sputter coating for nonconductive surfaces or indirect binding assays [6, 22]. Indirect binding assays require an additional growth step after material incubation to amplify individual binders, followed by serial dilution and replicated plating of library populations. Our new library incorporates an inducible DsRed-Express2 fluorescent protein to allow direct visualization and quantification of enrichment on sample surfaces to eliminate SEM sample preparation or indirect assays that consume time and laboratory supplies, and avoids potential errors caused by cell growth rate differences. After incubating PLA and sapphire pieces with induced libraries and subsequent washing, binding was quantified by measuring either fluorescent pixel coverage, or the number of CFUs from an indirect assay (Fig. 7). Similar trends were observed for each method of quantification, and in addition to significant differences between PLA and sapphire binding from the Round 2 library, the visualization assay also uncovered differences in Round 1 binding not found in the indirect assay (Fig. 7a).

While we demonstrate the enrichment of peptides with affinity for PLA in this work, and that the enriched peptide library is not purely "sticky" since it does not bind to sapphire with similar affinity (Fig. 8b), the primary goal of this work was to demonstrate how NGS and co-expression of fluorescent proteins can be used to enhance data analysis of display libraries in general, rather than to provide an optimized peptide sequence for binding PLA. To further optimize affinity and specificity of peptides for PLA, it could be useful to employ a constrained peptide library, as they have been shown to improve both affinity and specificity [19]. Specificity for PLA, for instance over other plastics, can also be improved by incorporation of negative sorting steps in future studies. Strategies including negative sorting have previously been shown to provide specificity over structurally similar biological targets [5, 17, 23], and are expected to analogously aid in biopanning peptides towards inorganic materials.

## Conclusions

Bacterial display is a valuable tool for rapidly screening peptide libraries. Our results demonstrate that the library generated for this study is diverse, contains a high proportion of valid inserts compared to phage libraries, and can be used successfully for material surface binding enrichment. Binding sequence motif analysis shows little spatial dependence and a general increase in the representation of hydrogen bond donors to interact with the polyester backbone of PLA. The DsRed-Express2 fluorescent protein provides an effective means for visualizing and quantifying adherent library members as an alternative method to indirect binding assays. Similar co-expression strategies with fluorescent proteins and substrate-specific peptides could also be employed for Förster resonance energy transfer (FRET) or other uses [24]. High-throughput next generation sequencing technology allows researchers to verify initial library diversity, gain a better understanding of the effects of selective pressure applied to library populations, and provide a baseline analysis for improving library design and application.

## Methods

### Bacterial strains, plasmids, materials and growth conditions

All experiments were performed with *E. coli* MC1061 F’ Electrocompetent cells strain SS320 (Lucigen; cat.no. 60512-2) [25]. Bacterial cells were routinely cultured in Luria-Bertani (LB) Miller broth (BD Difco) supplemented with 34 *μ*g/ml chloramphenicol (LB+Cm). Agar (15 g/L) was added for solid media and cultures maintained at 37^∘^*C*. Liquid cultures were grown in 14 ml culture tubes (Falcon) and shaken at 250 rpm. The eCPX peptide display scaffold was encoded on plasmid pBAD33-eCPX [16] and is available from Addgene. The plasmid was modified by replacing eCPX with eCPX3.0, which includes a P2X peptide displayed on the C-terminus [16]. Additional modification to the Addgene plasmid sequence included mutation of an *Sfi*I site upstream of the peptide insertion site to create a *Bsr*GI site for ease of library insertion. DsRed-Express2 was PCR amplified from commercial vector pCMV-DsRed-Express2 (Takara) and inserted directly upstream of eCPX. The backbone vector was constructed by co-inserting DsRed-Express2 and the modified eCPX 3.0 sequence into pBAD33-eCPX using *Sac*I and *Sal*I restriction sites to create pB33-dsRed-eCPX3.0 (Fig. 1).

To construct the library, the pB33-dsRed-eCPX3.0 backbone vector was digested with *Bsr*GI and *Xho*I and purified using DNA Clean and Concentrator kit (Zymo Research). A DNA library of peptide inserts in the form of NNS within the eCPX3.0 gene was commercially synthesized (GeneArt), similarly digested and ligated into the vector backbone using electroligase (New England Biolabs). A total of 1.5 *μ*g of newly assembled constructs were then electroporated into 250 *μ*l electrically competent cells in batches of 1 *μ*l DNA with 25 *μ*l cells using a 0.1 cm gap, pre-chilled electroporation cuvette, and pulsed at 1.8 kV, 10 *μ*F, and 100 *Ω*. The cuvette was rinsed three times with 1 ml warm SOC and transferred to a 125 ml vented flask. This process was repeated until the entire volume of pB33-dsRed-eCPX3.0 library was transformed (∼10 times). Approximately 70 ml warm SOC was added to the flask and incubated at 37^∘^*C* for 1 h. After recovery in SOC, the library transformants were added to 500 ml of warm LB+Cm for 4 h. The culture was then split into five flasks of 1 L LB+Cm and grown overnight. The following day, each liter was pelleted and resuspended in 10 ml LB+15% glycerol. From this, 1 ml frozen library stocks were prepared and stored at −80^∘^*C*, with each stock containing ∼10^11^ cells.

### PLA coupons

A 1 kg spool of 1.75 mm ’True Black’ PLA 3D Printer Filament from HATCHBOX *Ⓡ* was used to make 1 x 3 cm coupons for use in binding trials. PLA plastic filament for 3D printing is delivered in spools, and must be melted into thin pieces between smooth-surfaced materials. Two ∼4 cm pieces of PLA filament were placed side-by-side on the bottom of a Pyrex baking dish (which maintains a smooth surface and can withstand rapid changes in heat) and set onto a hot plate heated to level 6/10, or ∼90C. A 5 cm diameter sapphire (aluminum oxide) wafer was placed on top of the PLA filaments (Fig. 4). After several minutes, as the PLA melted, a small 200 gram weight was used to gently press the sapphire wafer, spreading the melting plastic underneath into a flat sample approximately 0.5 mm thick. Once fully melted, the Pyrex dish was immediately moved to a benchtop and allowed to cool for exactly 7 min. After cooling, a razor blade was used to carefully lift the PLA sample from the Pyrex dish and remove the sapphire wafer. Scissors sterilized with ethanol were used to cut the sample into 1 x 3 cm coupons for biopanning trials, 0.5 x 0.5 cm coupons for indirect assays, or 0.75 x 0.75 cm coupons for visualization. Sapphire wafer was also used to as an alternate material for comparison after binding trials (described below and depicted in Fig. 3). Sapphire has a smooth, characterized surface and can be cut to size using an etching tool.

### Biopanning

Biopanning rounds consist of several steps: 1) incubating the target material with the naive library culture, 2) washing unbound bacteria from the material surface, 3) placing the washed material with bound library members into fresh media, 4) allowing the bound members to amplify (resulting in the Round 1 library), and 5) repeating steps 1–4 for two total panning rounds. The pB33-dsRed-eCPX3.0 library was prepared for panning as previously described [6, 17, 26] with modifications depicted in Fig. 3 for the current study. In brief, peptide expression was induced overnight using 0.4% L-arabinose after 250 ml overnight stocks diluted 1:50 in LB+Cm reached an OD600 0.50–0.60. Induced cells were chilled on ice for 15 min and transferred to a sterilized crystalizing dish containing four 1 x 3 cm PLA samples sterilized with 70% ethanol. Samples were kept at 4^∘^*C* for 15 min with shaking to allow bacteria to bind. PLA coupons were removed from the library culture, briefly dipped into sterile phosphate buffered saline (PBS) to remove the bulk of unbound bacteria, and transferred to 20 ml of PBS supplemented with 1% Tween. Coupons were washed for 30 min at 330 rpm after incubation with the naive library, and for 1 h after incubation with the Round 1 library. After washing, bound cells were recovered by placing the PLA coupons into LB+Cm media supplemented with 2% D-Glucose (LB+Cm/Glu) and grown at 37^∘^*C* with shaking overnight. Fifty randomly selected colonies from Round 2 were sequenced using the pBAD Reverse universal primer (Genewiz).

### Visualization binding assay

In order to visually compare adhesion across samples after biopanning against PLA, 0.5 x 0.5 cm PLA coupons and sapphire pieces were incubated with induced 5 mL cultures of the naive library, sorting rounds, or negative control (which consists of a library member with an empty eCPX scaffold, i.e. no peptide). Cultures were prepared from freezer stocks grown overnight in LB+Cm. Fresh LB was inoculated 1:50 with the overnight stocks and brought to an OD of 0.5-0.6, then induced overnight with 0.4% arabinose as described above. PLA and sapphire pieces were incubated for 10 min with each culture at 230 rpm, then washed for an additional 10 min in fresh culture tubes containing 4 ml of PBS 1% Tween at 330 rpm to remove weakly bound cells. A final dip in 4 ml of fresh PBS ensured any unbound bacteria in the wash solution would be completely removed. Material samples were placed on microscope slides and visualized using an epifluorescent microscope (Nikon Eclipse TE2000-E confocal) at 40X and a red fluorescent filter (AT-TRITC/CY3). Samples were prepared independently in triplicate and ten images were captured from each sample and analyzed for the number of red fluorescent pixels using ImageJ [27]. Student’s T-tests were used to statistically verify differences in fluorescent pixel density.

### Indirect binding assay

Indirect binding assays were performed to quantify the number of cells bound to PLA and sapphire pieces as previously described [6]. In brief, incubation of sample materials with libraries and the negative control was the same as described above, however, after washing, the material samples were placed in 6 ml of fresh LB+Cm/Glu and grown for 1 h at 37^∘^*C* with shaking. Serial dilutions of amplified cells from each sample were plated on LB+Cm agar plates. Samples were prepared independently in triplicate and the averages and standard deviations determined. Student’s T-tests were used to statistically verify differences in relative binding results.

### Next generation sequencing

Three library culture samples were prepared for Illumina next generation sequencing: the naive library and libraries recovered after each of two rounds of biopanning. Plasmids were extracted from overnight cultures using a ZymoPure plasmid maxiprep kit (Zymo Research) and prepared for sequencing according to the Illumina 16S amplicon protocol using custom forward (AGTTCTGGCTTTCACCGCAG) and reverse (CCGTAGTACTGGTTTTTGTTGTAGTC) primers corresponding to eCPX scaffold regions adjacent to the peptide insert and run on a NextSeq500. Primers also included standard Illumina platform adapter overhangs and multiplex barcodes (not shown).

Illumina fastq files were analyzed using modified Matlab scripts based on the format presented in Matochko et al. (2012)[12]. Sequences were parsed into categories based on: 1) valid sequences: inserts that had both correct flanking sequences, were in the correct reading frame, contained no stop codons, and had all bases above the quality threshold (sequences with an Illumina quality score above Q20, i.e. bases that have a 99% or better chance of having been correctly assigned), 2) inserts containing nucleotides with incorrect reading frames, which resulted in a nonfunctional eCPX, 3) inserts in the correct reading frame that contained stop codons, and 4) empty eCPX scaffolds (scaffolds that did not contain any insert; Fig. 5). Valid sequences were then sorted to discover unique sequences. Additional sequence analysis was performed with in-house scripts, and the pLogo probability logo generator [18] was used to study sequence motifs.

## Supplementary information

**Additional file 1** **Figure S1** Amino acids represented within unique sequences. Amino acid representation of all unique sequences within each library **a** excluding and **b** including the effects of frequency of sequence occurrence for Naive (blue), Round 1 (orange) and Round 2 (grey). **Figure S2** Unique 15-mer sequence motifs from all libraries. Visualization of unique 15-mer sequence motifs calculated against background probabilities for *E. coli* K12 in **a** Naive **b** Round 1 and **c** Round 2 libraries, as generated by the pLogo software (http://plogo.uconn.edu) [18]. **Figure S3** Unique 15-mer sequence motifs from the Round 2 library and subsets of the Round 2 library. Visualization of unique 15-mer sequence motifs calculated against background probabilities for *E. coli* K12 in the Round 2 library: **a** all 15-mers **b** 40 most frequently occurring 15-mers and **c.** 10 most frequently occurring 15-mers, as generated by the pLogo software (http://plogo.uconn.edu) [18]. **Figure S4** Library growth curves. Growth curves over 6 h for the naive library, both sorting rounds, and the negative control after a 1:100 dilution from overnight cultures in fresh media. **Table S1** Raw count of total and unique sequences for all sequence categories of each library. **Table S2** Raw count and decimal of total sequences in library containing 0–4 cysteines, round by round.

## Data Availability

The NGS datasets and materials supporting the conclusions of this article are available from the corresponding author upon reasonable request.

## Abbreviations

NC: Negative control
NGS: Next generation sequencing
NL: Naive library
PLA: Polylactic acid
R1: Round 1
R2: Round 2
