# Supplementary material for: The next generation of biopanning: next gen sequencing improves analysis of bacterial display libraries
Source: BMC Biotechnol. 2019 Dec 21;19:100. doi: 10.1186/s12896-019-0577-8 (PMC6925417; doi:10.1186/s12896-019-0577-8)
Supplement: Supplementary file 1 — Additional file 1 Figure S1 Amino acids represented within unique sequences. Amino acid representation of all unique sequences within each library a excluding and b including the effects of frequency of sequence occurrence for Naive (blue), Round 1 (orange) and Round 2 (grey). Figure S2 Unique 15-mer sequence motifs from all libraries. Visualization of unique 15-mer sequence motifs calculated against background probabilities for E. coli K12 in a Naive b Round 1 and c Round 2 libraries, as generated by the pLogo software (http://plogo.uconn.edu) [18]. Figure S3 Unique 15-mer sequence motifs from the Round 2 library and subsets of the Round 2 library. Visualization of unique 15-mer sequence motifs calculated against background probabilities for E. coli K12 in the Round 2 library: a all 15-mers b 40 most frequently occurring 15-mers and c. 10 most frequently occurring 15-mers, as generated by the pLogo software (http://plogo.uconn.edu) [18]. Figure S4 Library growth curves. Growth curves over 6 h for the naive library, both sorting rounds, and the negative control after a 1:100 dilution from overnight cultures in fresh media. Table S1 Raw count of total and unique sequences for all sequence categories of each library. Table S2 Raw count and decimal of total sequences in library containing 0–4 cysteines, round by round. [file 12896_2019_577_MOESM1_ESM.pdf]

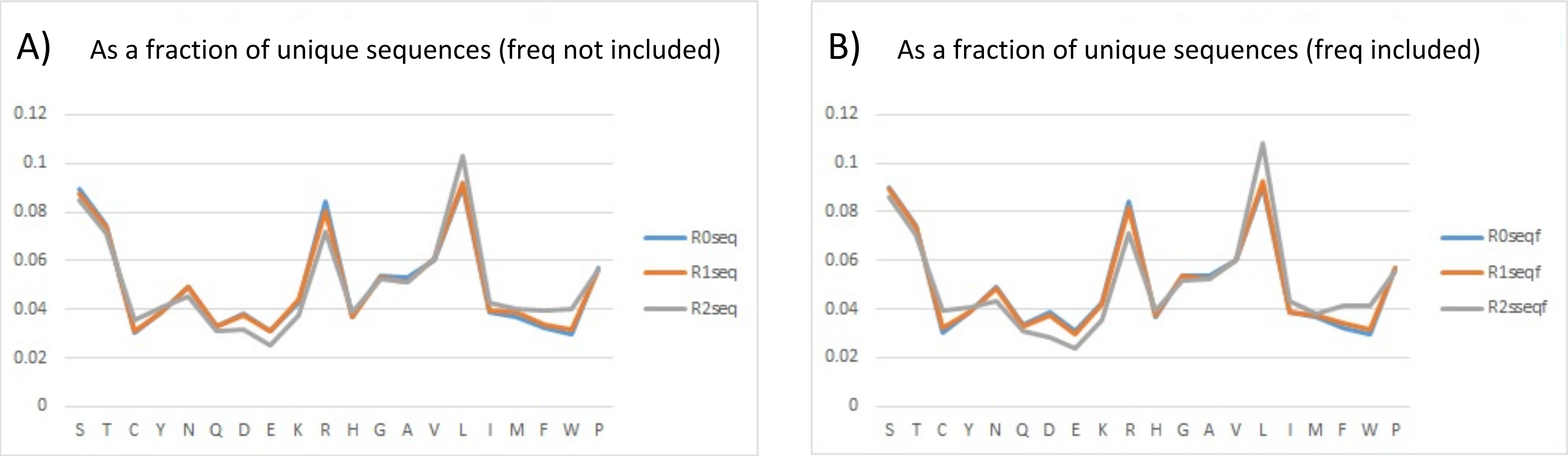

Supplementary Figure S1. Amino acid representation within all unique sequences within the library excluding (A) and including (B) the effects of frequency of sequence occurrence, for Round 0 (blue), Round 1 (orange) and Round 2 (grey).

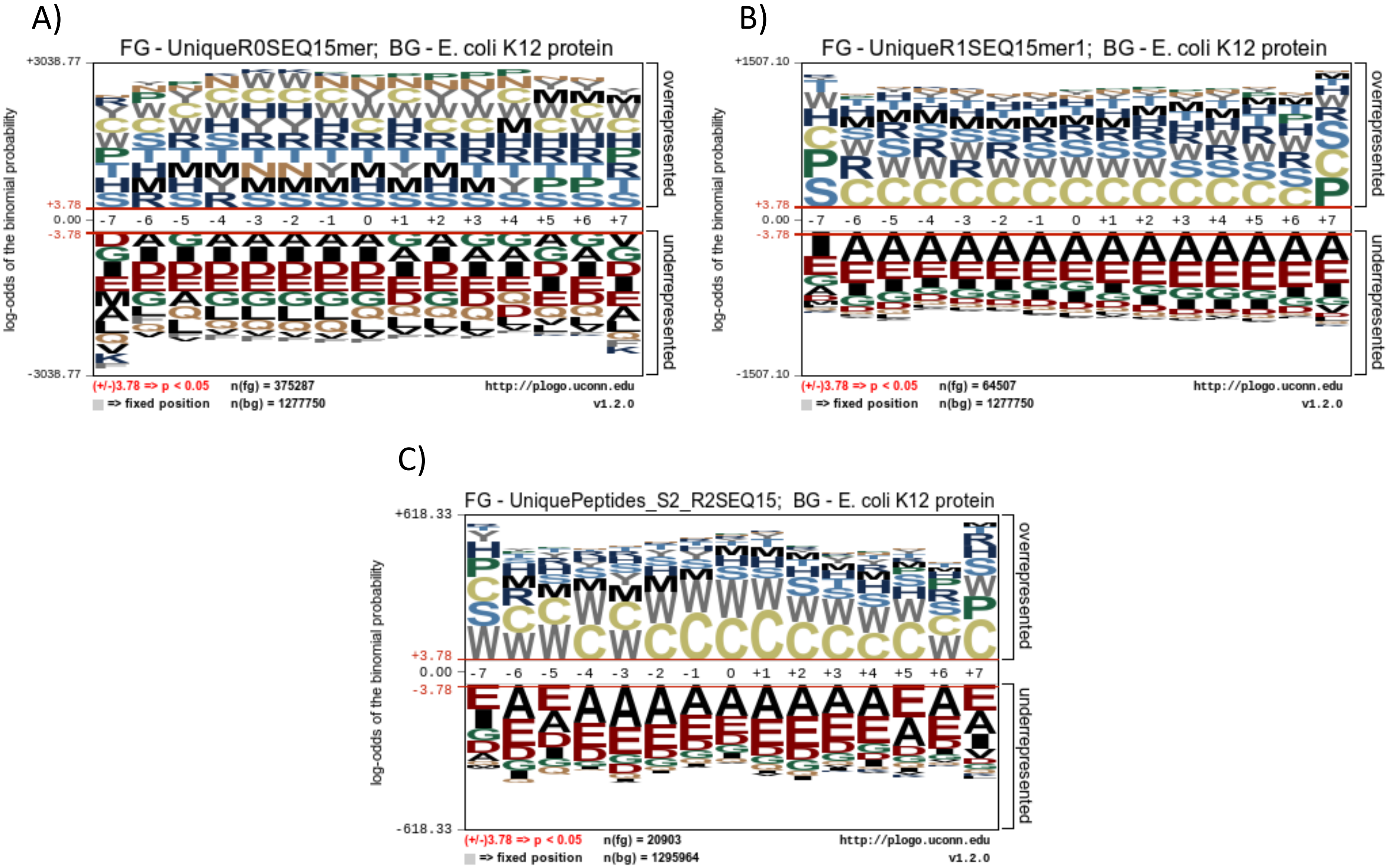

Supplementary Figure S2. Visualization of motifs of unique 15-mer sequences calculated against background probabilities for *E. coli* K12 in (A) Round 0 (B) Round 1 and (C) Round 2 libraries, as generated by the pLogo software (http://plogo.uconn.edu) of O'Shea *et al.* [21]

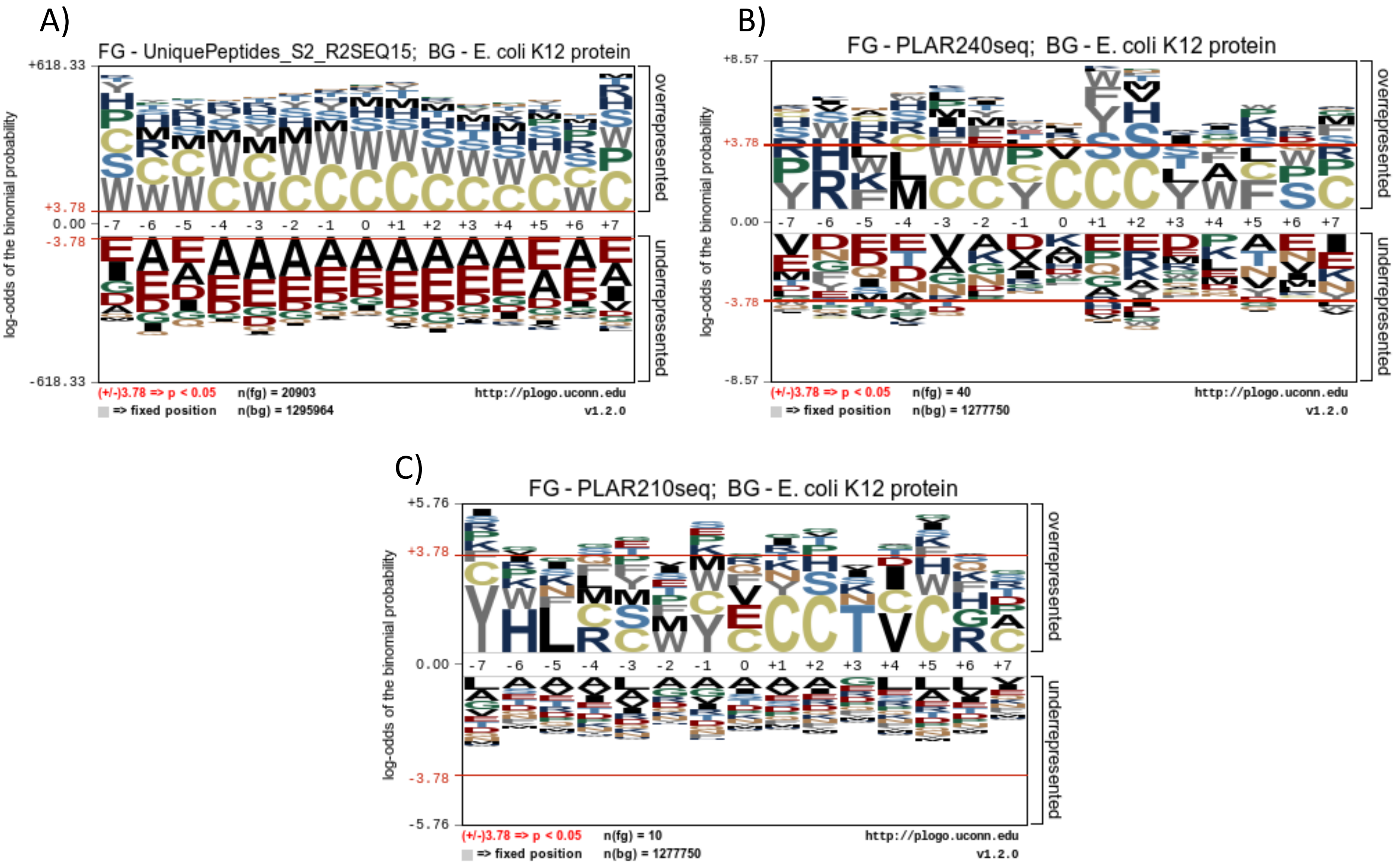

Supplementary Figure S3. Visualization of motifs of unique 15-mer sequences calculated against background probabilities for *E. coli* K12 in Round 2 library (A) all 15-mers (B) 40 most frequently occurring 15-mers and (C) 10 most frequently occurring 15-mers, as generated by the pLogo software (http://plogo.uconn.edu) of O'Shea *et al.* [21]

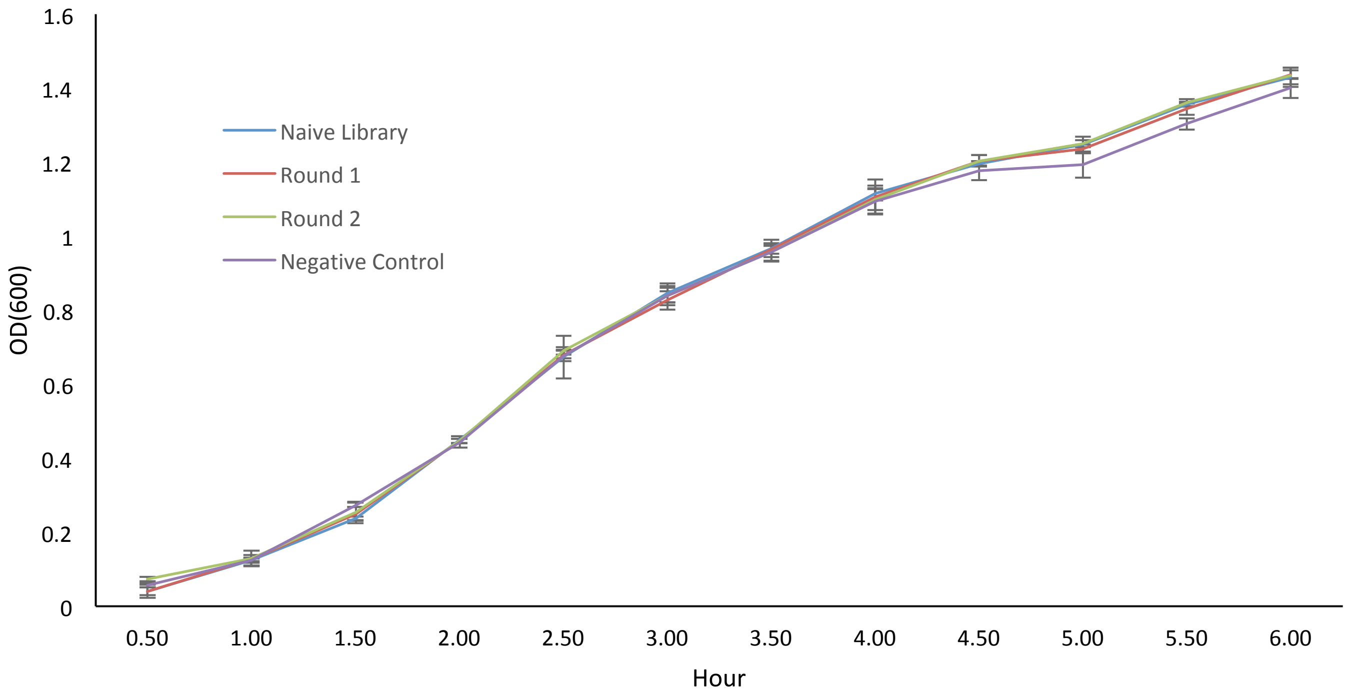

Supplementary Figure S4. Growth curves over 6 hours for the naive library, both sorting rounds, and the negative control after a 1:100 dilution from overnight cultures in fresh media.

		Valid Inserts	Frame Shift	Stop Codon	Empty Scaffold	Total # of Sequences
**Round 0**	**Total**	466,476	331,377	255,658	158,791	1,212,302
	**Unique**	396,641	313,601	224,685	---	---
**Round 1**	**Total**	591,400	350,783	299,598	176,237	1,418,018
	**Unique**	67,885	208,322	42,017	---	---
**Round 2**	**Total**	770,853	279,786	201,774	152,954	1,405,367
	**Unique**	21,931	133,599	7,966	---	---

Supplementary Table S1. Raw count of total and unique sequences for all sequence categories of each library.

		0 cysteines	1 cysteine	2 cysteines	3 cysteines	4 cysteines
**Round 0**	**raw count**	252165	116024	24846	3275	309
	**decimal**	0.635751	0.292516	0.062641	0.008257	0.000779
**Round 1**	**raw count**	42299	20649	4305	564	65
	**decimal**	0.623098	0.304176	0.063416	0.008308	0.000958
**Round 2**	**raw count**	12344	7809	1497	263	18
	**decimal**	0.562856	0.356071	0.06826	0.011992	0.000821

Supplementary Table S2. Raw count and decimal of total sequences in library containing 0-4 cysteines, round by round.
